# Trends in alcohol use among young people according to the pattern of consumption on starting university: A 9-year follow-up study

**DOI:** 10.1371/journal.pone.0193741

**Published:** 2018-04-09

**Authors:** Lucía Moure-Rodriguez, Carina Carbia, Eduardo Lopez-Caneda, Montserrat Corral Varela, Fernando Cadaveira, Francisco Caamaño-Isorna

**Affiliations:** 1 CIBER de Epidemiología y Salud Pública (CIBERESP), Department of Public Health, Universidade de Santiago de Compostela, Santiago de Compostela, Spain; 2 Department of Clinical Psychology and Psychobiology, Universidade de Santiago de Compostela, Santiago de Compostela, Spain; 3 Neuropsychophysiology Lab, Research Center on Psychology, School of Psychology, University of Minho, Braga, Portugal; Leibniz Institute for Prevention Research and Epidemiology BIPS, GERMANY

## Abstract

**Aim:**

To identify differences in Risky Consumption (RC) and Binge drinking (BD) trends in students who already followed these patterns of alcohol consumption on starting university and those who did not, and also to try to understand what leads students to engage in these types of behaviour at university.

**Material and methods:**

Cohort study among university students in Spain (n = 1382). BD and RC were measured with the Alcohol Use Disorders Identification Test at ages 18, 20, 22, 24 and 27 years. Multilevel logistic regression for repeated measures was used to calculate the adjusted Odds Ratios (ORs).

**Results:**

The prevalence rates of RC and BD were lower throughout the study in students who did not follow these patterns of consumption at age 18. For RC and BD, the differences at age 27 years, expressed as percentage points (pp), were respectively 24 pp and 15 pp in women and 29 pp and 25 pp in men. Early age of onset of alcohol use increased the risk of engaging in RC and BD patterns at university, for men (OR = 2.91 & 2.80) and women (OR = 8.14 & 5.53). The same was observed in students living away from the parental home for BD (OR = 3.43 for men & 1.77 for women). Only women were influenced by having positive expectancies for engaging in RC (OR = 1.82) and BD (OR = 1.96).

**Conclusions:**

The prevalence rates of both RC and BD at age 27 years were much higher among university students who already followed these patterns of consumption at age 18 years, with the differences being proportionally higher among women. Focusing on the age of onset of alcohol consumption and hindering access to alcohol by minors should be priority objectives aimed at preventing students from engaging in these patterns of alcohol consumption at university.

## Introduction

Alcohol is the most commonly consumed psychoactive substance worldwide [1]. Alcohol use often begins in early adolescence, a period when risky behaviours such as substance use are common [2]. Recent reports indicate that 47% of young Europeans have consumed alcohol at or before the age of 13 years [3]. In a Spanish survey, 21% of students reported being intoxicated in the 30 days prior to the evaluation, representing one of the highest mean rates among European countries [3]. Binge drinking (BD), a particular type of risky alcohol consumption, is defined as the consumption of large amounts of alcohol in a short period of time, with blood alcohol concentrations reaching up to 0.08 g/dl[4]. This pattern of consumption, is replacing among young people traditional alcohol use in Spain (one in four young people between the ages of 14 and 18 years partake in BD) [5]. BD has been associated with a wide range of negative consequences (e.g. neurocognitive deficits, other drug use, risky sexual behaviour), for both the drinkers themselves and also for others in their close environment [6,7].

In recent years, a great deal of scientific research has been conducted worldwide with the aim of understanding alcohol use in young people and designing effective prevention strategies. Identifying individual explanatory factors for this type of behaviour is crucial for obtaining accurate information about where we should focus our efforts. Some risk factors prevail among university students, although with some variations due to socio-cultural differences [8].

Age of onset of alcohol consumption is sometimes considered one of the most influential of these risk factors. Early age of onset has been associated with life-threatening outcomes, increased levels of RC throughout adolescence and greater risk of dependence during adulthood [9,10]. Most university students tend to drink more heavily than their non-student peers [11]. Although BD often starts during late adolescence, a large proportion of students seem to acquire this unhealthy pattern of consumption during their first years at university. In a study involving 1,894 first-year university students in the USA, Weitzman [12] found that 1 in 4 first started to partake in BD at university, probably because of environmental and temporal characteristics specific to the university environment [11].

Despite the importance of risky drinking patterns, longitudinal data regarding prevalence rates amongst university students -a population particularly at risk for alcohol-related problems- is still scarce. Thus, we wondered about the extent to which risky patterns of alcohol consumption in Spain are acquired at university or, conversely, are already established before university. We therefore decided to study the possible differences in long-term temporal trends in RC and BD in university students who had already started to follow these alcohol use patterns before going to university and those who began while attending university. On the basis of previous risk factors identified in this cohort [13], we also attempted to identify variables that induce university students to engage in RC and BD when they had not previously followed such patterns of consumption. The identification of such factors may help in the design of comprehensive prevention and intervention approaches adapted to an environment where alcohol tends to be widely available and prevalent [14].

## Materials and methods

### Design, population and sample

We carried out a cohort study among university students (Compostela Cohort 2005, Spain), between November 2005 and February 2015. We used cluster sampling to select the participants. Thus, at least one of the first-year classes was randomly selected from each of the 33 university faculties or departments (a total of 53 classes). The number of classes selected in each university faculty or department was proportional to the number of students. All students present in the class on the day of the survey were invited to participate in the study (n = 1382). A total of 99.06% of the students completed the questionnaire at the beginning of the study. Abstinent students were excluded from the association analysis, although the numbers are included in the sample description. This study was approved by the Bioethics Committee of the University de Santiago de Compostela. Subjects were informed both verbally and in written format (within the questionnaire) that participation was voluntary, anonymous, and the possibility to opt-out was available at any time. Subjects were informed that they were free to fill in or refuse to fill in the questionnaire. This procedure was approved by the Bioethics Committee.

### Data collection procedure

Two teams of researchers visited each first-year classroom in November 2005 and invited all students present in the class to participate in the study. Participants were evaluated via a self-administered questionnaire in the same classroom (1st questionnaire). In November 2007, the same team of researchers visited the third-year classroom in order to follow-up with the students. Participants were re-evaluated via a self-administered questionnaire (2nd questionnaire). The questionnaires were linked using birth date, sex, university department, and class. Students who provided a phone number in the first or second questionnaire were further evaluated by phone at 4.5-, 6.5-, and 9.0- year follow-ups (3rd, 4th and 5th questionnaires). On all five occasions, alcohol use was measured with the Galician validated version of the AUDIT [15,16]. In addition to the AUDIT, another questionnaire that asked about the potential factors associated with alcohol use was also administered (educational level and alcohol use by parents, alcohol-related problems and age of onset of alcohol use). One of the items in the second questionnaire specifically referred to alcohol-related expectancies. In this question, the students were required to rank 14 expectancies about the effects of alcohol (it adds fun, it helps me to socialize, to feel more relaxed, to forget about problems, to endure problems, it causes irritability, anxiety, depression, confusion, sleep-related problems, nervousness, aggression, loss of control, heaviness/drowsiness). This question was generated using items from a questionnaire previously administered to young Spanish adults [17]. More details about data collection are available in the following reference [13].

### Definition of variables

#### Independent variables

Several socio-demographic variables were considered: gender, place of residence (parental home/away from the parental home), and maternal educational level (primary school/high school/university). Four categories were defined for age of onset of alcohol use (after 16 years old, at age 16, at age 15, before the age of 15).

Finally, taking the number of positive and negative expectancies into account, a score ranging from 0 to 14 was generated (0 being the maximum of negative expectancies and 14 the maximum of positive expectancies). The scores were divided into tertiles.

#### Dependent variables

1. Risky consumption (RC). Dichotomous variable generated from the AUDIT score. A different cut-off value was established according to gender: = >5 for women; and = >6 for men. These cut-offs are recommended in the Galician validated version of the AUDIT [16].
2. Binge drinking (BD). This is a dichotomous variable generated from the third AUDIT question “How often do you have 6 or more alcoholic drinks per occasion?”, which was coded as follows: never = 0, less than once a month = 0, once a month = 1, once a week = 1, daily or almost daily = 1. The sensitivity and specificity of this question with this cut-off value are respectively 0.72 and 0.73, and the area under the curve is 0.767 (95% CI: 0.718–0.816) [18].

### Statistical analysis

We used multilevel logistic regression for repeated measures to obtain adjusted Odds Ratios (ORs) for independent variables from the final RC and BD models. Confidence intervals of 95% (95% CI) were calculated for both proportions and means. These models are more flexible than traditional models and therefore allow us to work with correlated data. This was the case here as the same subject was measured several times and the responses were strongly correlated, thus creating a dependency structure. The university faculty/department and classroom were considered random variables. We decided not to impute missing data, as analysis of the distribution of missing values enabled us to assume the non-existence of any patterns in the distribution of missing values. Maximal models were generated, including all theoretical independent variables according to the literature. Final models were generated from the maximal models. The nonsignificant independent variables were eliminated from this maximum model when the coefficients of the main exposure variables did not vary by more than 10% and the value of Akaike Information Criterion (AIC) decreased. Data were analyzed using Generalized Linear Mixed Models in SPSS v.20 statistical software.

## Results

The characteristics of the samples of women and men are summarized in Tables 1 and 2. There were no significant differences in any of these variables in either females or males.

**Table 1:** Characteristics of female initial sample and follow-up samples.

	Percentage or mean (95%CI)					
	Initial (18–19 years old) n = 992	2-year follow-up (20–21 years old) n = 669 (67.4%)	4 year follow-up (22–23 years old) n = 461 (46.5%)	6 year follow-up (24–25 years old) n = 266 (26.8%)	9 year follow-up (27–28 years old) n = 325 (26.8%)	p-value
**Maternal educational level**						
Primary school	41.8 (38.4–45.3)	44.2 (40.1–48.4)	43.1 (38.3–48.3)	47.3 (41.3–54.1)	45.7 (40.1–51.8)	
High school	33.6 (30.2–37.1)	30.5 (26.4–34.7)	30.6 (25.8–35.8)	26.5 (20.4–33.3)	28.1 (22.5–34.2)	
University	24.6 (21.2–28.1)	25.3 (21.3–29.6)	26.3 (21.4–31.4)	26.1 (20.1–32.9)	26.2 (20.7–32.4)	0.642
**Residence**						
In parental home	24.7 (22.1–27.5)	22.9 (19.7–26.1)	22.2 (18.5–26.0)	22.1 (18.1–26.1)	20.9 (16.5–25.1)	
Away from the parental home	75.3 (72.6–78.0)	77.1 (74.0–80.3)	77.8 (74.1–81.6)	77.9 (73.9–81.9)	79.1 (74.9–83.5)	0.720
**Positive expectations about alcohol**						
Low	37.1 (33.4–40.9)	37.5 (33.2–42.1)	36.5 (31.4–42.0)	36.5 (30.9–42.3)	37.9 (31.7–44.3)	
Medium	34.0 (30.3–37.8)	32.6 (28.3–37.3)	34.6 (29.4–40.1)	35.4 (29.8–41.1)	34.8 (28.6–41.2)	
High	28.9 (25.2–32.7)	29.9 (25.5–34.5)	28.9(23.7–34.4)	28.1 (22.5–33.8)	27.2 (21.0–33.6)	0.999
**Age of onset of alcohol use**						
After age 16	19.0 (16.5–21.8)	17.9 (14.9–21.3)	16.5 (13.0–20.5)	16.7 (12.1–22.5)	14.5 (10.5–19.2)	
Age 16	38.9 (35.6–42.2)	38.1 (34.1–42.2)	36.8 (32.0–41.7)	40.1(33.6–46.8)	36.6 (30.9–42.6)	
Age 15	25.6 (22.7–28.7)	25.9 (22.3–29.6)	26.5 (22.2–31.1)	26.4 (20.8–32.7)	28.3 (23.0–34.0)	
Before age 15	16.5 (14.0–19.7)	18.1 (15.0–21.5)	20.3 (16.4–24.5)	16.7 (12.1–22.5)	20.7 (16.0–25.9)	0.438
**Binge drinking**^a^						
Never	61.2 (58.2–64.3)	61.3 (57.7–65.1)	59.0 (54.7–63.7)	59.4 (53.8–65.5)	60.0 (54.8–65.4)	
Less than once a month	20.9 (17.8–23.9)	20.9 (17.3–24.7)	23.4 (19.1–28.1)	22.2 (16.5–28.3)	22.5 (17.2–27.9)	
Monthly	9.8 (6.7–12.8)	9.1 (5.5–12.9)	9.1 (4.8–13.8)	9.8 (4.1–15.9)	9.8 (4.6–15.3)	
More frequently	8.2 (5.1–11.2)	8.7 (5.1–12.5)	8.5 (4.1–13.2)	8.6 (3.0–14.8)	7.7 (2.5–13.1)	0.999
**AUDIT: Total (mean)**	5.4 (5.2–5.7)	5.6 (5.1–5.8)	5.6 (5.2–6.0)	5.6 (5.0–6.1)	5.3 (4.9–5.8)	0.884

^a^ Question 3 of the Alcohol Use Disorders Identification Test (AUDIT).

**Table 2:** Characteristics of male initial sample and follow-up samples.

	Percentage or mean (95%CI)					
	Initial (18–19 years old) n = 371	2-year follow-up (20–21 years old) n = 206 (55.5%)	4-year follow-up (22–23 years old) n = 139 (37.5%)	6-year follow-up (24–25 years old) n = 81 (21.8%)	9-year follow-up (27–28 years old) n = 90 (24.2%)	p-value
**Maternal educational level**						
Primary school	32.0 (26.5–37.8)	35.8 (28.4–43.3)	41.6 (32.8–50.8)	43.0 (31.6–54.8)	41.6 (31.5–53.5)	
High school	27.6 (22.1–33.3)	27.4 (19.9–34.9)	25.5 (16.8–34.7)	24.1 (12.7–35.8)	27.0 (16.8–38.9)	
University	40.3 (34.8–46.0)	36.8 (29.3–44.3)	32.8 (24.1–42.0)	32.9 (21.5–44.7)	31.5 (21.3–43.4)	0.449
**Residence**						
In the parental home	29.7(25.1–34.5)	27.8 (21.9–34.1)	28.8 (21.6–36.4)	31.6 (23.9–40.6)	28.9 (20.0–38.3)	
Away from the parental home	70.3 (65.7–75.1)	72.2 (66.3–78.5)	71.2 (64.0–78.9)	68.4 (60.7–77.4)	71.7 (62.2–80.5)	0.949
**Positive expectations about alcohol**						
Low	29.7 (23.7–36.0)	33.0 (25.1–41.0)	34.2 (25.0–44.3)	35.4 (25.3–46.4)	31.6 (20.3–43.7)	
Medium	38.0 (32.0–44.4)	30.7 (22.9–38.8)	31.7 (22.5–41.8)	32.3 (22.2–43.4)	30.4 (19.0–42.5)	
High	32.3 (26.3–38.7)	36.3 (28.5–44.4)	34.2 (25.0–44.3)	32.3 (22.2–43.4)	38.0 (26.6–50.0)	0.705
**Age of onset of alcohol use**						
After age 16	18.1 (12.5–24.1)	16.8 (9.2–24.7)	15.5 (6.9–25.5)	16.4 (6.0–29.7)	18.2 (7.8–30.3)	
Age 16	36.9 (31.2–42.8)	41.0 (33.5–49.0)	44.0 (35.3–54.0)	50.7 (40.3–64.0)	48.1 (37.7–60.1)	
Age 15	21.6 (15.9–27.5)	20.2 (12.7–28.2)	21.6 (12.9–1.6)	23.9 (13.4–37.2)	20.8 (10.4–32.8)	
Before age 15	23.4 (17.8–29.4)	22.0 (14.4–30.0)	19.0 (10.3–9.0)	9.0 (0.0–22.3)	13.0 (2.6–25.1)	0.381
**Binge drinking**^a^						
Never	39.1 (34.0–44.7)	43.2 (36.4–50.6)	42.4 (34.5–51.7)	46.9 (37.0–58.9)	45.6 (35.6–56.5)	
Less than once a month	25.3 (20.2–31.0)	20.4 (13.6–27.8)	21.6 (13.7–30.8)	21.0 (11.1–33.0)	21.1 (11.1–32.1)	
Monthly	12.7 (7.5–18.3)	14.6 (7.8–22.0)	13.7 (5.7–22.9)	17.3 (7.4–29.3)	15.6 (5.6–26.5)	
More frequently	22.9 (17.8–28.6)	21.8 (15.0–29.2)	22.3 (14.4–31.6)	14.8 (4.9–26.8)	17.8 (7.8–28.8)	0.905
**AUDIT: Total (mean)**	7.8 (7.2–8.4)	7.4 (6.6–8.2)	7.3 (6.4–8.2)	6.5 (5.4–7.6)	7.1 (6.0–8.2)	0.784

^a^ Question 3 of the Alcohol Use Disorders Identification Test (AUDIT).

At the beginning of the study, the rates of prevalence of RC and BD among females were 51.5% (95% CI: 48.4–54.6) and 17.9% (95% CI: 15.6–20.3), while among males the respective rates were 58.0% (95% CI: 52.9–63.0) and 35.6% (95% CI: 30.7–40.5). As shown in Tables 3 and 4, the percentage of subjects partaking in RC or BD was always lower in females than in males at ages 20, 22, 24 and 27. The prevalence decreased in those students who already engaged in RC or BD before going to university, particularly for BD among women (see Table 3). For all subjects, regardless of gender or the age of onset of alcohol use, the greatest decrease in the prevalence of both RC and BD always occurred between the ages of 22 and 24 years (Table 3).

**Table 3:** Percentages of subjects partaking in risky consumption and binge drinking at age 20, 22, 24 and 27 years, among subjects already partaking in each of these consumption patterns at age 18.

	Females								Males							
	Risky consumption				Binge drinking				Risky consumption				Binge drinking			
	Age				Age				Age				Age			
	20	22	24	27	20	22	24	27	20	22	24	27	20	22	24	27–28
**Maternal educational level**																
Primary school	74.3 (136)	58.9 (95)	18.0 (61)	30.3 (76)	36.2 (47)	25.8 (31)	0 (21)	21.4 (26)	87.9 (33)	65.4 (26)	28.6 (14)	33.3 (18)	81.8 (22)	52.9 (17)	27.3 (11)	36.4 (11)
High school	79.6 (113)	59.0 (78)	20.0 (40)	26.0 (50)	52.5 (40)	32.0 (25)	7.1 (14)	23.5 (17)	79.3 (29)	85.0 (20)	12.5 (8)	54.5 (11)	84.2 (19)	80.0 (15)	20.0 (5)	50.0 (8)
University	84.2 (95)	70.3 (74)	17.5 (40)	40.4 (52)	59.4 (32)	44.0 (25)	7.1 (14)	11.5 (14)	90.0 (50)	68.8 (32)	47.4 (19)	47.6 (21)	63.6 (33)	64.7 (17)	44.4 (9)	27.3 (11)
**Residence**																
In parental home	72.3 (65)	53.3 (45)	16.7 (30)	27.6 (29)	47.6 (21)	25.0 (12)	10.0 (10)	19.6 (6)	82.1 (28)	73.7 (19)	45.5 (11)	27.3 (11)	68.4 (19)	75.0 (12)	42.9 (7)	16.7 (6)
Away from home	80.3 (279)	64.4 (202)	18.9 (111)	32.9 (149)	48.0 (98)	34.8 (69)	2.6 (39)	0 (51)	87.1 (85)	71.7 (60)	32.3 (31)	48.7 (39)	76.8 (56)	63.2 (38)	31.6 (19)	41.7 (24)
**Positive expectancies about alcohol**																
Low	72.6 (62)	47.6 (42)	12.5 (24)	25.0 (32)	25.0 (16)	20.0 (10)	0 (6)	16.7 (6)	87.5 (16)	53.8 (13)	22.2 (9)	57.1 (7)	87.5 (8)	16.7 (6)	20.0 (5)	75.0 (4)
Medium	77.7 (121)	57.4 (94)	21.8 (55)	27.1 (69)	45.7 (35)	26.9 (26)	0 (14)	10.5 (19)	83.3 (36)	69.2 (26)	54.5 (11)	62.5 (16)	72.7 (22)	71.4 (14)	50.0 (6)	28.8 (7)
High	79.8 (124)	71.3* (87)	17.0 (47)	42.4* (59)	58.0 (50)	36.4 (33)	5 (20)	13.0 (23)	87.5 (48)	77.4 (31)	33.0 (18)	27.3 (22)	72.7 (33)	77.3* (22)	25.0 (12)	35.7 (14)
**Age of onset of alcohol use**																
After age 16	76.5 (34)	72.2 (18)	22.2 (9)	7.7 (13)	28.6 (7)	60.0 (5)	50 (2)	0 (4)	100 (11)	66.7 (9)	20.0 (5)	33.3 (6)	71.4 (7)	50.0 (6)	33.3 (3)	50.0 (4)
Age 16	79.8 (119)	56.0 (84)	17.3 (52)	29.5 (61)	46.4 (28)	18.8 (16)	9.1 (11)	8.3 (12)	87.8 (41)	83.9 (31)	50.0 (22)	40.0 (25)	70.8 (24)	72.2 (18)	42.9 (14)	42.9 (14)
Age 15	81.9 (105)	66.2 (67)	13.0 (46)	31.6 (57)	50.0 (40)	35.7 (28)	0 (18)	23.8 (21)	82.6 (23)	56.2 (16)	22.2 (9)	33.3 (9)	66.7 (15)	66.7 (9)	40.0 (5)	0 (5)
Before age 15	75.0 (84)	63.6 (66)	28.1 (32)	40.0 (45)	51.2 (43)	35.5 (31)	0* (17)	15.8 (9)	91.2 (34)	75.0 (20)	0 (5)	62.5 (8)	82.1 (28)	68.8 (16)	0 (3)	33.3 (6)
**Total of subjects 95% Cl:** **Lower** **Upper**	**78.8** **74.5** **83.7**	**62.3** **56.3** **68.4**	**18.4** **12.0** **24.8**	**32.0**+ **25.2** **38.9**	**47.9** **38.9** **56.9**	**33.3** **23.1** **43.6**	**4.1** **0.0** **9.6**	**17.5**+ **7.7** **27.4**	**86.0** **79.6** **92.3**	**72.2** **62.3** **82.0**	**35.7** **21.2** **50.5**	**44.0**+ **30.2** **57.8**	**74.7** **68.4** **84.5**	**66.0** **52.9** **79.1**	**34.6** **16.3** **52.9**	**36.7**+ **19.4** **53.9**

Note: The total number of subjects is shown between brackets.

*Significant differences among categories of exposition. χ^2^ test, p<0.05.

^+^Significant differences among ages. χ^2^ test, p<0.05.

**Table 4:** Percentages of subjects partaking in risky consumption and binge drinking at age 20, 22, 24 and 27 years, among subjects who did not partake in each of these consumption patterns at age 18.

	Females								Males							
	Risky consumption				Binge drinking				Risky consumption				Binge drinking			
	Age				Age				Age				Age			
	20	22	24	27	20	22	24	27	20	22	24	27	20	22	24	27
**Maternal educational level**																
Primary school	21.7 (157)	20.6 (102)	6.2 (64)	6.9 (72)	8.1 (246)	8.4 (166)	6.7 (104)	3.3 122)	35.9 (39)	29.0 (31)	5.0 (20)	15.8 (19)	16.0 (50)	22.5 (40)	0 (23)	11.5 (26)
High school	25.8 (89)	22.6 (62)	0 (30)	4.9 (41)	11.1 (162)	17.4 (115)	3.6 (56)	0 (74)	34.6 (26)	40.0 (15)	9.1 (11)	7.7 (13)	19.4 (36)	30.0 (20)	7.1 (14)	12.5 (16)
University	26.0 (53)	21.7 (46)	6.9 (29)	12.1 (33)	12.5 (136)	11.6 (95)	0 (55)	2.8 (71)	29.2 (24)	30.8 (13)	14.3 (7)	28.6 (7)	22.0 (41)	39.3 (28)	23.5* (17)	11.8 (17)
**Residence**																
In parental home	16.1 (87)	22.8 (57)	3.0 (33)	5.1 (39)	5.3 (131)	11.1 (90)	1.9 (53)	0 (62)	31.0 (29)	23.8 (21)	0 (16)	13.3 (15)	13.2 (38)	10.7 (28)	5.0 (20)	5.0 (20)
Away from home	27.4* (234)	20.6 (155)	5.5 (91)	8.3 (108)	11.6* (415)	12.2 (288)	4.9 (163)	2.9 (206)	34.9 (63)	38.5 (39)	13.0 (23)	16.0 (25)	20.7 (92)	39.3* (61)	11.4 (35)	15.0 (40)
**Positive expectancies about alcohol**																
Low	17.5 (154)	16.0 (106)	2.9 (68)	5.1 (78)	5.5 (200)	6.5 (138)	2.3 (86)	2.9 (104)	25.6 (43)	32.1 (28)	5.0 (20)	22.2 (18)	15.7 (51)	20.0 (35)	0 (24)	9.5 (21)
Medium	29.9 (67)	32.6 (46)	12.0 (25)	12.5 (32)	11.1 (153)	16.7 (114)	7.6 (66)	1.2 (82)	47.4 (19)	25.0 (12)	12.5 (8)	0 (8)	21.2 (33)	25.0 (24)	23.1 (13)	29.4 (17)
High	25.0 (48)	26.7 (30)	0 (19)	5.0 (20)	13.9* (122)	13.1* (84)	2.2 (46)	3.6 (56)	41.2 (17)	40.0 (10)	0 (5)	12.5 (8)	18.8 (32)	52.6* (19)	9.1 (11)	0* (16)
**Age of onset of alcohol use**																
After age 16	25.7 (70)	12.5 (48)	3.4 (29)	0 (27)	7.2 (97)	6.6 (61)	5.6 (36)	0 (36)	38.9 (18)	55.6 (9)	16.7 (6)	25.0 (8)	27.3 (22)	33.3 (12)	0 (8)	10.0 (10)
Age 16	30.4 (102)	28.6 (63)	10.3 (39)	12.5 (40)	14.0 (193)	17.6 (131)	5.0 (80)	2.2 (89)	50.0 (30)	40.0 (20)	16.7 (12)	16.7 (12)	25.5 (47)	33.3 (33)	25.0 (20)	8.7 (23)
Age 15	42.2 (45)	48.3 (29)	7.1 (14)	19.0 (21)	12.7 (110)	15.4 (78)	0 (42)	1.8 (57)	41.7 (12)	55.6 (9)	0 (7)	28.6 (7)	20.0 (20)	43.8 (16)	0 (11)	27.3 (11)
Before age 15	19.0 (21)	26.7* (15)	0 (6)	8.3 (12)	9.7 (62)	12.0 (50)	14.3 (21)	7.9 (38)	50.0 (4)	0 (2)	0 (1)	0 (2)	20.0 (10)	33.3 (10)	0 (3)	0 (4)
**Total of subjects 95% Cl:** **Lower** **Upper**	**24.0** **19.4** **28.6**	**21.0** **15.6** **26.5**	**4.8** **1.1** **8.5**	**7.5**+ **3.2** **11.7**	**10.0** **7.5** **12.5**	**11.8** **8.6** **15.1**	**4.1** **1.5** **6.8**	**2.2**+ **0.5** **4.0**	**33.7** **24.0** **43.4**	**33.3** **21.4** **45.3**	**7.7** **0.0** **16.1**	**15.0**+ **3.9** **26.1**	**18.3** **11.7** **24.9**	**30.3** **20.8** **39.9**	**9.1** **1.5** **16.7**	**11.7**+ **4.5** **18.9**

Note: The total number of subjects is shown between brackets.

*Significant differences among categories of exposition. χ^2^ test, p<0.05.

^+^Significant differences among ages. χ^2^ test, p<0.01.

**Fig 1:**
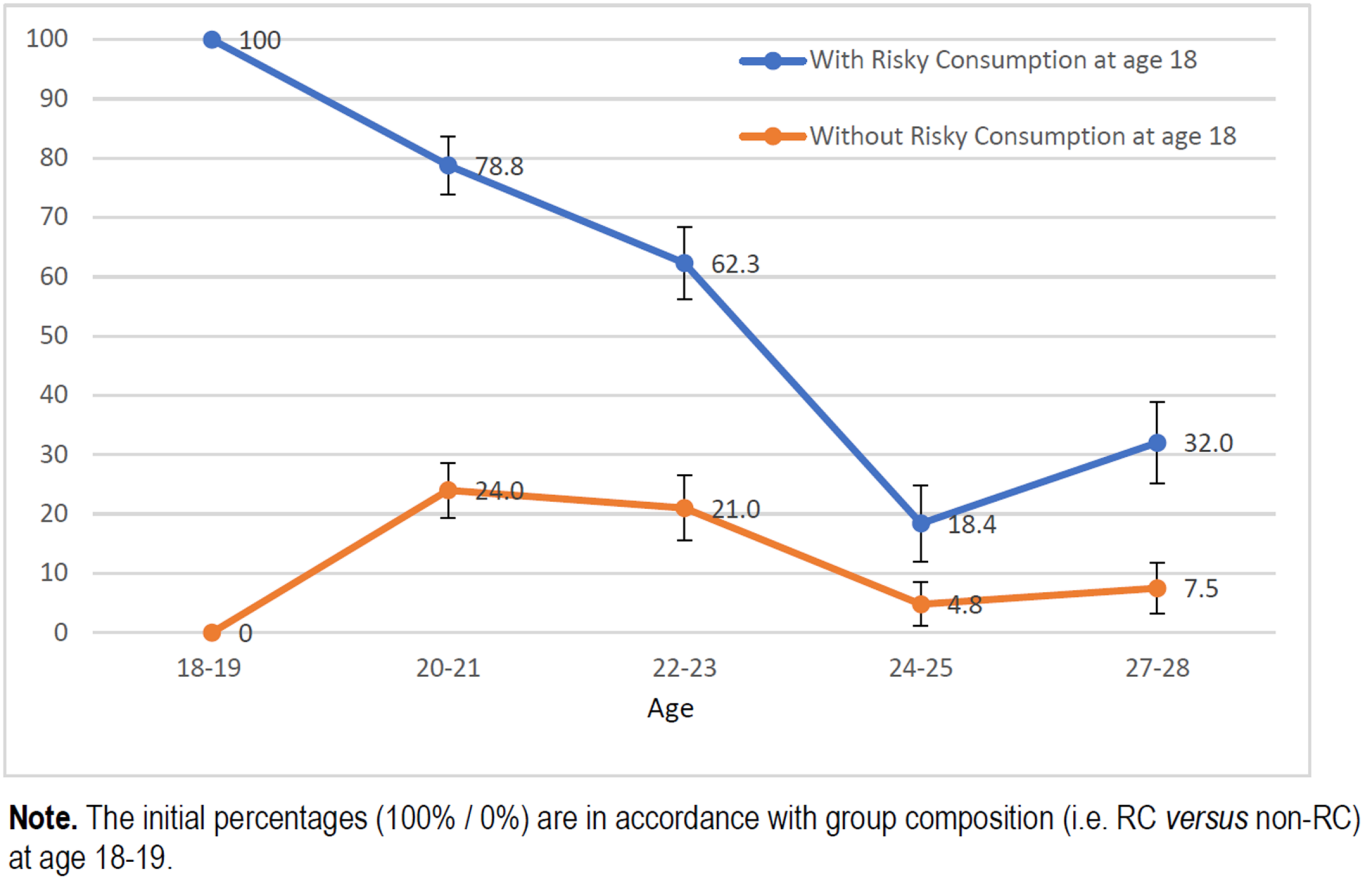
Trends in prevalence of risky consumption (%) among women who already partook and who did not partake in risky consumption at age 18–19.

**Fig 2:**
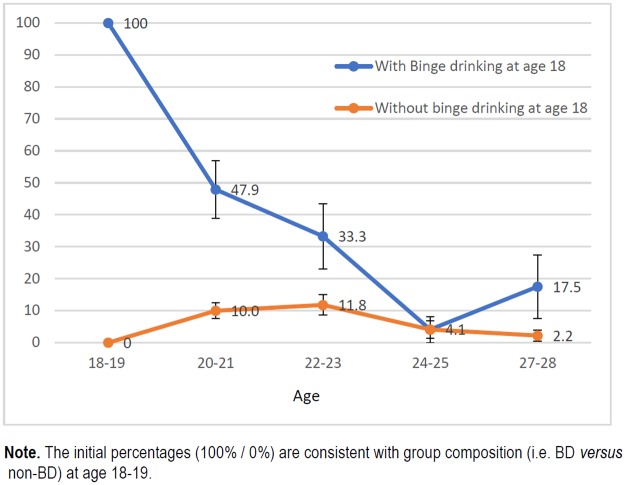
Trends in prevalence of binge drinking (%) among women who already partook and who did not partake in binge drinking at age 18–19.

**Fig 3:**
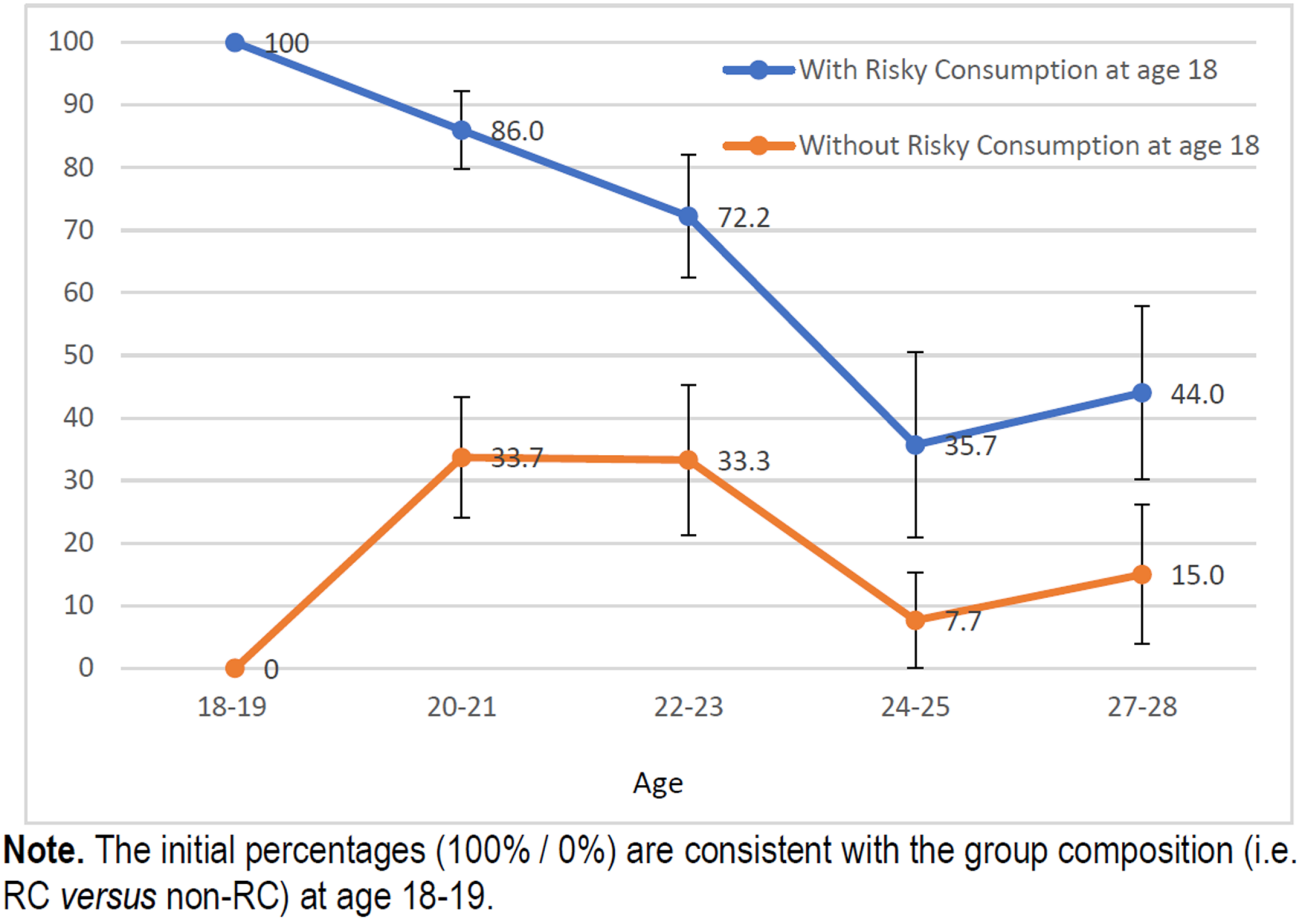
Trends in prevalence of risky consumption (%) among men who already partook and who did not partake in risky consumption at age 18–19.

**Fig 4:**
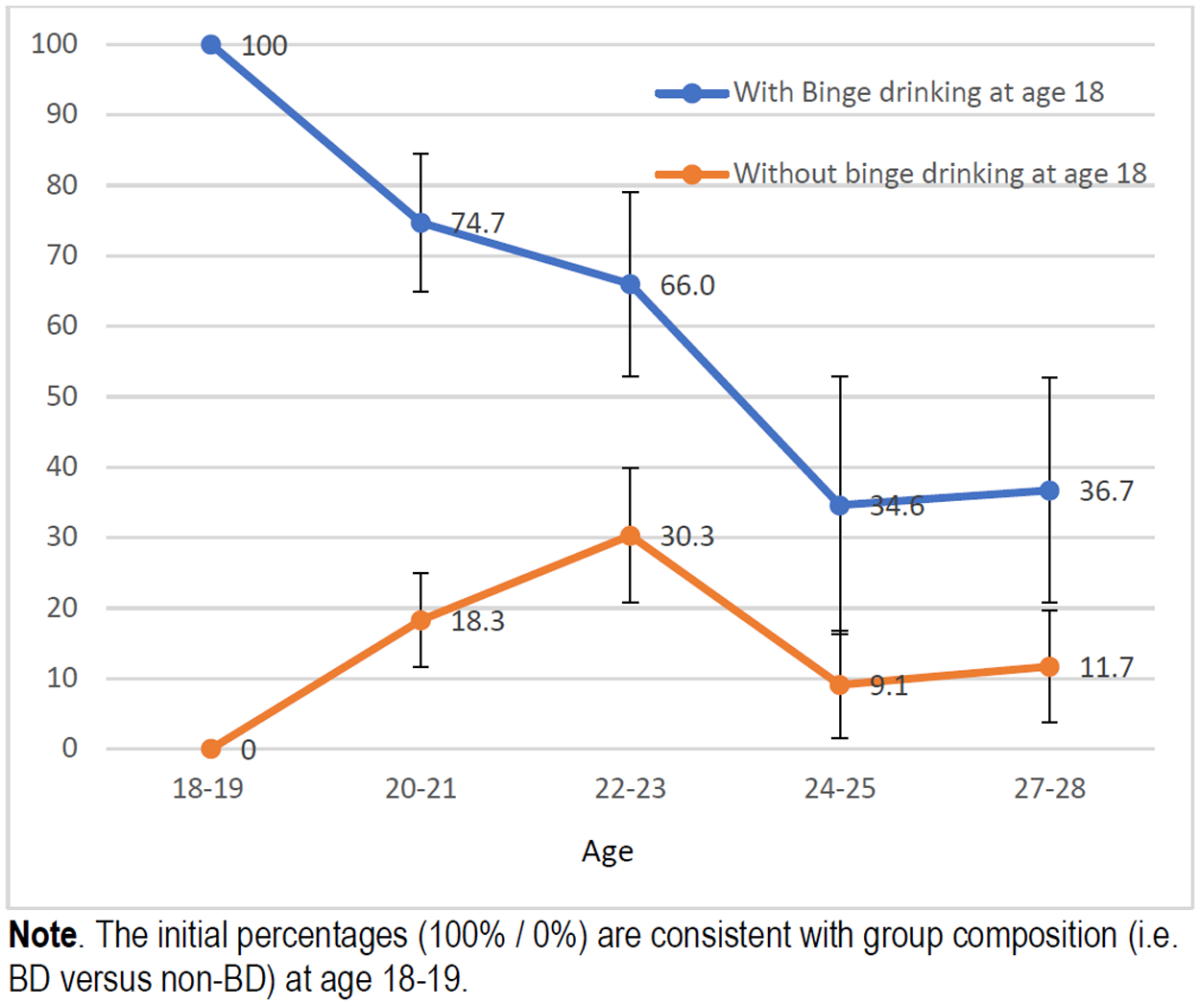
Trends in prevalence of binge drinking (%) among men who already partook and who did not partake in binge drinking at age 18–19.

Figs 1, 2, 3 and 4 show the trends in the prevalence of RC and BD during the study period for students who had followed and students had not followed RC and BD patterns of alcohol use at age 18. The prevalence rates were significantly lower throughout the study in students who did not follow these consumption patterns at the beginning of study than in those who already partook in these types of behaviour. At age 27 years the differences for RC and BD were respectively 24 and 29 pp for females and 15 and 25 pp for males.

In relation to the factors associated with engaging in RC or BD after starting university, the multivariate analysis presented in Table 5 reveals that age of drinking onset is one of the most influential factors for both women (OR = 8.14 for RC and OR = 5.53 for BD) and men (OR = 2.91 for RC and OR = 2.80 for BD), with the risk being significantly higher among women. In the final logistic regression models, the categories “at age 15” and “before the age of 15” were grouped taking into account that the OR for those starting alcohol at age 15 and those starting before the age of 15 was the same.

**Table 5:** Influence of different variables on risky consumption and binge drinking in subjects who did not partake in either of these consumption patterns at age 18–19 years.

	Multivariate analysis^a^			
	Odds ratio (95%CI)			
	Risky Consumption		Binge Drinking	
	Females^¥^	Males^£^	Females*	Males^+^
**Age of onset of alcohol use**				
At age 17 or elder	1	1	1	1
At age 16	8.14 (4.35–15.25)	2.91 (1.41–6.00)	5.53 (2.71–9.65)	2.80 (1.36–5.74)
At age 15 and before age 15	6.63 (3.32–13.24)	2.52 (1.07–5.92)	4.69 (2.28–9.28)	2.25 (1.09–5.94)
**Residence**				
In parental home			1	1
Away from the parental home			1.77 (1.05–3.00)	3.43 (1.60–7.38)
**Positive expectancies about alcohol**				
Low	1		1	
Medium	1.82 (1.08–3.06)		1.96 (1.18–3.25)	
High	1.48 (0.75–2.95)		1.80 (1.05–3.08)	
**Age of participants**				
20–21 years	1	1	1	1
22–23 years	0.98 (0.55–1.75)	0.94 (0.46–1.93)	1.24 (0.79–1.95)	2.08 (1.08–4.04)
24–25 years	0.09 (0.05–0.17)	0.15 (0.04–0.53)	0.40 (0.19–0.83)	0.47 (0.17–1.27)
27–28 years	0.15 (0.08–0.31)	0.30 (0.11–0.81)	0.24 (0.11–0.50)	0.60 (0.24–1.50)

^a^ Adjusted by all variables included in the column.

^¥^ Number of subject measures included in the model: 693.

^£^ Number of subject measures included in the model: 231.

* Number of subject measures included in the model: 1250.

^+^ Number of subject measures included in the model: 334.

Among women, positive expectancies about alcohol consumption increased the risk of engaging in RC and BD at university (OR = 1.82 and OR = 1.96 respectively) while in men no such influence was observed. Living outside the family home increased the risk of starting BD at university in both men (OR = 3.43) and women (OR = 1.77). In both women and men, age of participants was a protective factor for engaging in BD (OR = 0.24 and OR = 0.60) and RC (OR = 0.15 and OR = 0.30) at university. We measured paternal and maternal alcohol use and educational level. None of these variables showed association with RC or BD.

## Discussion

The study findings show that the rates of prevalence of both Risky consumption (RC) and Binge drinking (BD) at age 27 years were much greater among university students who already followed these consumption patterns at age 18 years, particularly among women. The age of onset of alcohol consumption proved the most important risk factor for students who had not previously partaken in RC or BD on starting university to engage in these alcohol use patterns, with the risk being significantly higher among women. Living outside the family home also increased the possibility that university students, both male and female, would start BD at university, which highlights the relevance of campus drinking culture [19,20]. Finally, only women who did not follow these patterns of consumption before attending university were influenced by having positive expectancies regarding alcohol consumption.

Previous studies have shown that risky alcohol consumption is described by an inverted-U curve that peaks in the early 20s [21,22], as demonstrated in this cohort [13]. However, the trend appears to differ depending on the “drinking status” at the beginning of the university period. Thus, for students who did not engage in RC or BD patterns of consumption before going to university, the distribution of these patterns was described by a bell-shaped curve. On the contrary, among those students who already partook in RC or BD before starting university, there was a steady decrease in the prevalence after late adolescence, in both men and women. Women who already followed a BD pattern of alcohol use, showed a decrease in consumption by more than 50% in only two years. Nonetheless, despite the clear reduction in excessive alcohol consumption, this group exhibited the highest prevalence throughout the follow-up period. The most plausible interpretation for the trend showed by this group is that we were actually observing the maximum peak (18–19 years) and the progressive upward trend occurred before reaching this age, as suggested by Bewick [23].

The prevalence rates of RC and BD, as we already mentioned, were lower during the study in those students who did not follow these patterns of consumption at the beginning of the study than in those students who did partake in these types of behaviour. The prevalence of RC at age 27 years were 4.2% in females and 2.9% in males, while for BD the prevalence rates were 7.8% and 3.9%. These results show that engaging in these patterns of consumption at an early age has a greater effect on alcohol consumption at age 27 years in women than in men.

A common trend in all groups, regardless of gender, consumption pattern or the age of onset, is the marked decrease in the prevalence of the patterns of consumption between ages of 22 and 24 years. This may be due to the fact that at the age of 24 years most of the participants had completed their university studies and began working. According to many authors this vital period is accompanied by the acquisition of adult roles with new responsibilities, causing young people to abandon certain types of behaviour, such as the patterns of alcohol consumption under consideration [24].

Regarding gender differences, rates of consumption have always been higher in men than in women. Although in young people gender differences in alcohol consumption are tending to decrease [25], in most European countries consumption is still generally more prevalent among men [25–27]. In the present study, we found that such gender differences were much more pronounced for BD, regardless of the age of onset, which may be partly due to the fact that the cut-off point we used to identify BD practitioners did not differentiate between genders. Thus, the prevalence of BD may have been underestimated in women, in whom the amount of alcohol ingested to be considered BD is lower [4].

Although the rates were much lower at the end of the study, the prevalence of risky consumption observed in 27-year-olds remained high, contradicting the traditional idea that these types of consumption are inherent in, but limited to young adulthood [28,29]. The rates were especially high among those who already followed these types of patterns before entering the university. It has been demonstrated that heavy drinking during adolescence is associated with neurocognitive alterations (e.g. inhibitory control, working memory) that at the same time might contribute to perpetuating the heavy drinking behaviour [30,31]. If we consider that BD peaks during the early twenties and then gradually declines, this result is particularly important as a significant number of young people seem to maintain these patterns during emerging adulthood, probably constituting a special at-risk subgroup for further alcohol escalation and other psychiatric disorders in adulthood [32]. Our findings appear to be consistent with those of longitudinal studies carried out in other countries, which also conclude that a considerable number of people maintained patterns of excessive drinking during adulthood [33]. The high level of youth unemployment caused by the economic crisis in Spain [34] may be delaying the assumption of adult roles and thus contribute to the continuance of risky consumption observed in the present study.

Age of drinking onset was the most important factor influencing those university students who did not previously partake in RC or BD and who then engaged in them at university. We found that even in subjects who did not follow the RC or BD patterns at 18 years old but had begun to consume alcohol at an early age (before the age of 16), the risk of engaging in BD or RC from age 19 onwards was between 5 and 8 times higher in women and more than 2 times higher in men, than if the age of onset of alcohol use was older (at 17 years old or older). The age of onset has already been shown to have an important influence on the pattern of alcohol consumption during late adolescence in the study cohort [13]; however, the present study highlights the fact that the age of onset is not only a risk factor for engaging in excessive drinking during late adolescence but also during university years (students aged 19 and over), emphasizing the long-term influence of this important risk factor [35].

The multivariate analysis revealed that living away from the family home increased the risk that university students, regardless of gender, would engage in BD at age 19 years or older, which may be associated with the reduction in parental monitoring and living in a more permissive environment [36,37]. This was not observed for RC. The differences regarding the influence of the place of residence on both types of consumptions may be partly explained by a greater normalization of non-BD consumptions among Mediterranean cultures. Moreover, peer pressure within the campus environment is likely to promote binge drinking behaviour, as peers may directly provide alcohol, act as role models or make BD appear common and acceptable. [38]

Among those women who did not follow these patterns of consumption at the beginning of the university period, having positive expectancies regarding alcohol use seems to increase the risk of engaging in both RC (OR = 1.82) and BD (OR = 1.96) at university. Although our findings are consistent with the results of several studies (greater effect of positive expectancies in women (e.g., [39,40]), other researchers have observed this effect among males (e.g. [41,42]). Thus, more studies are needed to clarify this point. Numerous studies have shown that relative to moderate drinkers, young people with risky patterns of alcohol consumption tend to have higher expectations of the positive effects of alcohol (e.g. social facilitation) and lower expectations of the negative effects (e.g. risks and aggression) [43]. In fact, positive alcohol expectancies have been shown to be a risk factor for initiation of alcohol consumption and further escalation in alcohol use [44], especially for BD [45–49]. Conversely, negative expectancies may be a protective factor for heavy drinking in young people [50,51]. This factor (diminishing positive and enhancing negative beliefs) may be a key aspect in developing prevention and intervention strategies [51,52].

Finally, the age of the subjects acts as a protective factor, reducing the risk of students engaging in both patterns of consumption throughout their time at university. These results are consistent with the figures that represent the trends in both patterns of consumption, where the prevalence tends to decline as the subjects become older. This is also confirmed by the fact that these types of behaviour are characteristic of young rather than older adults [53].

There are four main limitations to this study: 1) As in other cohort studies, the loss of subjects at follow-up can lead to selection bias. Nonetheless, there were no significant differences among participants throughout the study period, suggesting the absence of such bias; 2) Information bias, which is always likely when a self-reported data is used. To minimize this, we used the AUDIT, a questionnaire that has been validated internationally among adolescents and young adults; 3) The third question of the AUDIT does not allow for gender differences, so that the prevalence of BD in women is underestimated in this study, by not taking into account women who drink 5 drinks on a single occasion. However, this only affects descriptive outcomes and not the statistical findings; and 4). The question about expectancies was not specifically validated and therefore expectancies may not have been correctly measured.

In conclusion, engaging in RC and BD before the age of 18 years leads to much higher prevalence of these patterns of alcohol use throughout young adulthood in university students. Having started drinking alcohol at a younger age increases the risk of engaging in these patterns during the time at university. Living outside the family home increases the risk of starting BD from the age of 19 years, and positive expectancies increase the likelihood of women engaging in RC and BD at this age. In light of these findings, it is essential to implement preventive measures that hinder access to alcohol by minors (before going to university) as well as environmental strategies within the university environment.
